# The Relationship Between Nurses’ Emotional Competence and Evidence-Based Nursing: A Scoping Review

**DOI:** 10.3390/nursrep15040124

**Published:** 2025-04-04

**Authors:** Dora Ribeiro Machado, Manuel Morais Brás, Assunção Laranjeira de Almeida, Carlos Vilela

**Affiliations:** 1 School of Medicine and Biomedical Sciences, University of Porto, Rua Jorge de Viterbo Ferreira, 228, 4050-313 Porto, Portugal; 2 RISE-Health, Nursing School of Porto, Rua Dr. António Bernardino de Almeida 830/844/856, 4200-072 Porto, Portugal; laranjeira.almeida@ua.pt (A.L.d.A.); carlosvilela@esenf.pt (C.V.); 3 LiveWell Research Center, Polytechnic Institute of Bragança, Campus de Santa Apolónia, 5300-253 Bragança, Portugal; manuel-bras@ipb.pt; 4 School of Health, University of Aveiro, Campus Universitário de Santiago, Edifício 30, 3810-193 Aveiro, Portugal

**Keywords:** nurses, emotional intelligence, evidence-based nursing, scoping review, nursing, emotional competences

## Abstract

**Background:** Emerging evidence suggests that emotions significantly influence clinical decision-making among healthcare professionals. Given that evidence-based nursing (EBN) relies heavily on clinical reasoning, and emotions play a critical role in shaping its quality, exploring the relationship between emotional competence and EBN is essential. **Objective:** This scoping review aims to map and synthesize existing knowledge on the relationship between nurses’ emotional competence and EBN, while identifying research methodologies and integration challenges. **Methods:** Following the Joanna Briggs Institute (JBI) methodology and PRISMA-ScR guidelines, a scoping review was conducted. The search strategy included studies from databases such as Scopus and CINAHL, as well as grey literature. Eligibility criteria included primary and secondary research articles in Portuguese, English, Spanish, and French, published since 1990, focusing on the relationship between emotional competence and EBN in nurses. Data were synthesized thematically. **Results:** Of 751 publications identified, 11 met the inclusion criteria. Three themes emerged: (1) the relationship between emotional competence and EBN in different healthcare contexts; (2) research methodologies used; and (3) integration challenges and suggestions. Findings suggest that nurses with higher emotional competence are more likely to adopt safer, evidence-based practices, facilitating EBN implementation and improving care quality and safety. **Conclusions:** The evidence highlights the importance of integrating emotional intelligence and EBN in nursing education and practice. Combined educational programs are recommended to enhance professional safety, performance, and well-being. Future research should further explore this relationship to develop practice models that reconcile emotional competencies with evidence-based nursing.

## 1. Introduction

Evidence-based nursing (EBN) and emotional intelligence (EI) are pivotal elements in contemporary nursing practice. While EBN focuses on integrating the best available evidence into clinical decision-making, EI emphasizes the ability to recognize, understand, and manage emotions in oneself and others [1]. Despite their individual significance, the interplay between these two concepts has not been thoroughly examined, particularly in terms of how emotional competence influences the implementation of EBN. This scoping review aims to map the existing knowledge on this relationship, addressing key research questions to provide a comprehensive understanding of its implications for nursing practice.

The foundations of evidence-based nursing (EBN) can be traced back to the pioneering work of Florence Nightingale in the 19th century [2], with further contributions from Archie Cochrane and the development of evidence-based medicine at McMaster University [3,4]. These historical milestones laid the groundwork for modern EBN, which is defined as the conscious, clear, and careful use of knowledge derived from theory and evidence in decisions about care provided to individuals or groups, while considering their needs and preferences [4,5].

EBN is based on a 5-step model: (i) understanding how to formulate a clinical question; (ii) identifying and gathering the most relevant scientific evidence; (iii) critically appraising the evidence; (iv) integrating the evidence into nursing practice, considering the patient’s needs, preferences, and personal values, to make clinical decisions; and (v) evaluating the results [6].This model underscores the importance of combining evidence with clinical expertise and patient preferences, ensuring that care is both effective and patient-centered [4].

By allowing the integration of evidence into care practice, EBN increases the quality and safety of care [7] and professional satisfaction [8], reduces difficulties faced by nurses in their clinical practice, facilitates autonomous interventions [9], reduces care costs [10], improves client outcomes [11], promotes a more informed and effective approach to clinical decision-making, with reduced subjectivity and bias [12], and is essential for holistic nursing practice [13].

Despite the widely acknowledged importance of EBN, its implementation remains suboptimal in various healthcare contexts [10,14,15,16,17], primarily due to barriers such as insufficient resources, time constraints, and inadequate training [14,18]. These challenges highlight the need for a deeper exploration of the factors influencing EBN adoption, including the role of emotional competence.

Emotions play a crucial role in clinical decision-making, influencing both cognitive reasoning and social behavior [19,20]. Emotional intelligence (EI), defined as the ability to recognize, understand, express, and regulate one’s own emotions and those of others, has been shown to enhance nurses’ ability to manage stress, communicate effectively, and make informed clinical decisions [21,22]. EI is operationalized through various models, including ability, trait, and mixed models, each offering unique perspectives on how emotional competence can be developed and applied in practice [23,24,25,26,27].

Connor et al. [27] argue that the choice of models and measurement instruments should be guided by the study’s specific objectives. For example, when the goal is to assess competencies, the ability model is recommended because it captures maximal performance. Similarly, if the aim is to examine how emotional intelligence relates to decision-making, the ability model is also preferred, given the importance of peak performance in that context.

However, the relationship between emotional competence and EBN remains underexplored. While studies have demonstrated the impact of emotions on clinical decision-making [19,20,28,29,30], there is a paucity of research on how emotional competence specifically influences the integration of evidence into nursing practice. Addressing this gap is essential for enhancing both the quality of care and the well-being of nursing professionals.

This scoping review aims to map the existing knowledge on the relationship between nurses’ emotional competence and evidence-based nursing. By addressing the following research questions, we aim to provide a comprehensive understanding of this relationship and its implications for nursing practice:

- What is the relationship between nurses’ emotional competence and evidence-based practice in different healthcare contexts?
- Which research methodologies are most frequently used to study the relationship between emotional competence and evidence-based practice among nurses?
- What are the main challenges identified in the implementation of evidence-based practices that may be related to nurses’ emotional competence?

## 2. Materials and Methods

This scoping review was conducted following the Joanna Briggs Institute (JBI) methodology [31] and adhered to the Preferred Reporting Items for Systematic Reviews and Meta-Analyses extension for Scoping Reviews (PRISMA-ScR) guidelines [32]. The review process involved five key stages: (i) defining the search strategy; (ii) identifying relevant studies; (iii) selecting studies; (iv) data extraction; and (v) presentation and discussion of results. The review protocol was pre-registered on the Open Science Framework (OSF) at https://doi.org/10.17605/OSF.IO/TNH7B (accessed on 25 March 2025).

### 2.1. Search Strategy

The search strategy was designed to identify both published and unpublished studies examining the relationship between emotional competence and evidence-based practice (EBP) in nurses. A preliminary search was conducted across multiple databases, including MEDLINE/PubMed, LILACS, Scopus, Web of Science, Google Scholar, Epistemonikos, Campbell Collaboration, FigShare, OSF, BMJ Open, and JBI Review Registry, to ensure no prior reviews or protocols on the same topic existed. The selection of these databases was justified by their comprehensive coverage of scientific literature in health and social sciences, which is highly relevant to the study’s focus.

### 2.2. Inclusion and Exclusion Criteria

The eligibility criteria for study inclusion were defined using the PCC framework (Population, Concept, Context), as recommended by the JBI methodology [31].

#### 2.2.1. Inclusion Criteria

- **Population**: Studies focusing on active nurses, irrespective of their specialty or work setting.
- **Concept**: Studies addressing emotional competence and evidence-based practice, including evidence-based nursing, evidence-based nursing practice, and evidence-based decision-making. Emotional competence was operationalized using competency models of emotional intelligence, as these models are widely accepted in scientific and academic circles.
- **Context**: All healthcare settings, including public and private institutions, primary and hospital care, and elderly care facilities, were included, provided they involved direct or remote patient contact. No geographical or temporal restrictions were applied.
- **Language and Publication Date**: The review included primary and secondary research articles published in Portuguese, English, Spanish, and French, from 1990 onward. This timeframe was selected because it marks the introduction of the emotional intelligence model by Mayer and Salovey, which is foundational to the study of emotional competence.

#### 2.2.2. Exclusion Criteria

- Studies that included nurses alongside other healthcare professionals without differentiating the results.
- Studies involving nursing students unless the results could be separated from those of practicing nurses, as the focus of this review is on clinical practice rather than educational settings.
- Studies that included nurses without at least a bachelor’s degree.
- Studies conducted in non-healthcare settings, even if they involved patient contact.
- Studies simulating care practices, such as those conducted in classroom settings.
- Studies focusing on emotional competence in the context of learning rather than practice.
- Opinion articles, editorials, commentaries, and narrative reviews.

Language Restriction Justification: The review was limited to studies published in Portuguese, English, Spanish, and French. This decision was based on the authors’ proficiency in these languages, ensuring accurate interpretation and analysis of the studies. While this may have excluded some relevant studies in other languages, these four languages represent a significant portion of the global nursing literature, particularly in regions where research on emotional competence and evidence-based nursing is most active. This approach maintains the review’s rigor and feasibility while minimizing the risk of misinterpretation due to language barriers.

### 2.3. Search Process

The search process was conducted in three stages. First, a preliminary search was performed in MEDLINE/PubMed, APA PsycInfo, Scopus, Web of Science, CINAHL Complete, and Cochrane databases to identify relevant articles. Keywords and index terms from the titles and abstracts of these articles were used to develop a comprehensive search strategy. Second, the identified keywords and descriptors were combined and adapted to the specific requirements of each database or repository. Boolean operators (e.g., “OR”, “AND”) and the “*” wildcard tool were used to refine the search and capture variations of the terms (see Appendix A). Finally, the reference lists of all included studies were reviewed to identify additional relevant publications.

The search was conducted across the following electronic databases: CINAHL Complete (via EBSCO), MEDLINE Complete (via EBSCO), Nursing & Allied Health Collection: Comprehensive (via EBSCO), Cochrane Central Register of Controlled Trials (via EBSCO), MedicLatina (via EBSCO), APA PsycInfo, Psychology and Behavioral Sciences Collection, Scopus, and Web of Science.

Grey literature was also searched using sources such as Grey Matters, WorldCat, Google Scholar, Agency for Healthcare Research and Quality (AHRQ), OpenGrey.EU, Repositórios Científicos de Acesso Aberto de Portugal (RCAAP), Estudo Geral - Repositório Digital da Universidade de Coimbra, and Ria – institutional repository of the University of Aveiro. The most recent search was conducted in October 2024.

Importance of Grey Literature: Grey literature was included in the search strategy to mitigate publication bias and ensure comprehensive coverage of the topic. Grey literature, which includes unpublished studies, theses, dissertations, and reports, often provides valuable insights not available in traditional peer-reviewed journals. By incorporating grey literature, this review aims to capture a broader range of perspectives and evidence, enhancing the robustness of the findings regarding the relationship between emotional competence and evidence-based practice.

### 2.4. Data Extraction and Quality Assessment

Data from the included studies were extracted by two independent reviewers using a standardized data extraction tool. The tool captured the following information: authors, publication date, title, study type, methodology, instruments used, sample size, country where the study was conducted, study context and objectives, main results and conclusions, operationalization of the relationship between EBN and emotional competence, and challenges related to the implementation of EBP. Disagreements between reviewers were resolved through discussion, and a third reviewer was not required.

The methodological quality of the included studies was assessed using the Joanna Briggs Institute (JBI) Critical Appraisal Checklist. This tool was selected for its suitability in evaluating diverse study designs, including qualitative, quantitative, and mixed-methods research. The checklist was used to assess key aspects such as study design, methodology, and reporting of results. However, as this is a scoping review focused on mapping the existing literature, the quality assessment was primarily used to provide an overview of the methodological rigor of the included studies rather than to exclude studies based on quality. This approach aligns with the purpose of scoping reviews, which emphasize breadth over depth of evidence. The findings from the quality assessment were used to inform the discussion of the results, highlighting potential limitations in the evidence while maintaining the review’s focus on mapping the breadth of the literature.

### 2.5. Data Synthesis

The data were synthesized thematically, with key themes and patterns identified in relation to the research questions. The thematic analysis was conducted using a structured approach, with two independent reviewers coding the data and resolving discrepancies through discussion. For example, one of the emerging themes was the role of emotional competence in facilitating interprofessional collaboration, which was consistently highlighted across multiple studies. The themes were derived inductively from the data, ensuring they accurately reflected the content of the included studies.

### 2.6. Ethical Considerations

This review is based on publicly available literature and does not involve human subjects; therefore, ethical approval was not required.

### 2.7. Identification and Selection of Relevant Studies

Following the search, all identified citations were uploaded to Rayyan (Rayyan QCRI, version 2023) for citation management and duplicate removal. After a pilot test, titles and abstracts were screened by two independent reviewers (DM and MB) against the inclusion criteria. Potentially relevant studies were retrieved in full text, and citation details were imported into the JBI System for Unified Management, Assessment, and Review of Information (JBI SUMARI) [33].

Full texts of the selected studies were assessed in detail by two independent reviewers to confirm eligibility. Reasons for excluding studies were documented and are reported in this review. Disagreements between reviewers were resolved through discussion, and a third reviewer was not required. The results of the search and selection process are presented in the PRISMA-ScR flow diagram [32].

### 2.8. Critical Appraisal

A critical appraisal of individual studies was not conducted, as the primary objective of this scoping review was to map the available literature and identify gaps for future research. This approach aligns with the purpose of scoping reviews, which focus on providing a broad overview of existing research rather than evaluating its rigor in depth. However, the lack of critical appraisal is acknowledged as a limitation, as it does not allow for distinguishing between studies of varying methodological quality.

## 3. Results

**Figure 1:**
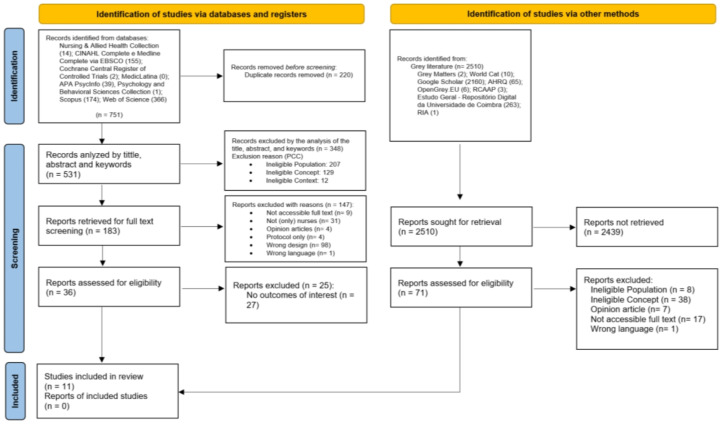
PRISMA-ScR flowchart, study identification and inclusion process.png.

Figure 1 outlines the article selection process for this review, following the PRISMA-ScR flow diagram guidelines. A total of 751 potentially relevant studies were identified from the selected databases. After removing 220 duplicates, 531 studies remained. Following a screening of titles, abstracts, and keywords, 348 studies were excluded. A further 147 articles were eliminated after a full-text review, as they did not meet the inclusion criteria. Of the 36 studies that advanced to the next stage, 25 were excluded for failing to address either the primary or secondary research questions. Additionally, 2510 studies were identified from the grey literature, but none met the criteria for inclusion. Ultimately, 11 studies were selected for this review.

Table 1 provides a summary of the studies included in this review, detailing their characteristics, methodologies, and key findings. The results are organized into four thematic areas, reflecting the main aspects explored in the literature:

1. Impact of Emotional Competence on Quality and Safety of Care: Adams and Iseler [34] and Fujino et al. [35] found a strong link between emotional intelligence and quality of care indicators. Their results suggest that nurses with higher EI are more likely to adhere to evidence-based protocols, leading to improved patient safety and care quality. For detailed findings, see Table 2.
2. Role of Emotional Intelligence in Clinical Reasoning and Decision-Making: Emotional intelligence was shown to significantly enhance clinical reasoning and decision-making, as highlighted by Bahmanpour et al. [36] and Hutchinson et al. [37]. This skill enables nurses to manage emotions effectively, communicate with patients and colleagues, and make patient-centered decisions [38,39], even in ethically challenging situations [40]. Further details are provided in Table 3.
3. Emotional Competence and Interprofessional Collaboration in EBN Implementation: Clarke et al. [41] and Hov et al. [42] demonstrated that emotional intelligence fosters interprofessional collaboration, which is crucial for successfully implementing EBP. Nurses with high EI exhibit better communication skills and adaptability, facilitating teamwork and the integration of EBP into routine care. See Table 4 for a comprehensive overview.
4. Emotional Competence in Cultural and High-Complexity Contexts: Al-Hamdan et al. [43] and Williams et al. [44] explored the impact of emotional intelligence on professional performance in culturally diverse and high-complexity settings. Their findings revealed that EI supports cultural adaptation, enhances digital communication, and enables quick decision-making under pressure, particularly in telemedicine environments. These results highlight the critical role of emotional intelligence in modern healthcare. Detailed results are presented in Table 5.

**Table 1:** Summary of included studies and methodologies.

Author, Year	Study Title	Study Type	Objectives	Methodological Approach	Main Themes
Btoush et al., 2024 [38]	The relationship between emotional intelligence, self-efficacy, and clinical decision-making among critical care nurses in Jordan	Quantitative	To assess EI’s role in clinical decisions	Cross-sectional, descriptive, and correlational	Emotional competence in clinical decision-making
Clarke et al., 2023 [41]	Advanced nurse and midwife practitioners’ experience of interprofessional collaboration when implementing evidence-based practice into routine care: An interpretative phenomenological analysis	Qualitative	To explore collaboration in EBP implementation	Interpretive phenomenological analysis	Interprofessional collaboration in EBP
Williams et al., 2019 [44]	Telemedicine intensive care unit nursing interventions to prevent failure to rescue	Qualitative	To explore tele-ICU nurses’ interventions	Interpretive qualitative analysis	Emotional competence in high-complexity contexts
Zaki et al., 2018 [39]	The effect of emotional intelligence program on decision making style	Quantitative	To evaluate an EI training program	Quasi-experimental	Emotional competence in decision-making
Bahmanpour et al., 2018 [36]	Critical thinking in clinical nursing: a content analysis	Qualitative	To understand critical thinking in nursing	Phenomenological	Emotional competence in clinical reasoning
Hutchinson et al., 2017 [37]	The use of emotional intelligence capabilities in clinical reasoning and decision-making_A qualitative, exploratory study	Mixed (qualitative phase)	To explore EI’s role in clinical reasoning	Constructivist thematic analysis	Emotional competence in clinical reasoning
Al-Hamdan et al., 2016 [43]	Correlating Emotional Intelligence and Job Performance Among Jordanian Hospitals’ Registered Nurses	Quantitative	To investigate EI’s impact on job performance	Descriptive, cross-sectional correlational	Emotional competence in professional performance
McLemore et al., 2015 [40]	Calculus Formation: Nurses’ Decision-Making in Abortion-Related Care	Qualitative	To describe decision-making in ethically complex contexts	Descriptive	Emotional competence in ethical decision-making
Adams & Iseler, 2014 [34]	The Relationship of Bedside Nurses’ Emotional Intelligence With Quality of Care	Quantitative	To examine the link between EI and care quality	Cross-sectional, correlational	Emotional competence in quality of care
Fujino et al., 2014 [35]	The relationship between characteristics of nursing performance and years of experience in nurses with high emotional intelligence	Quantitative	To explore EI’s impact on nursing performance	Cross-sectional, correlational	Emotional competence in professional performance
Hov et al., 2009 [42]	Being a nurse in nursing home for patients on the edge of life	Qualitative	To understand nurses’ experiences in end-of-life care	Phenomenological, hermeneutic	Emotional competence in end-of-life care

In this review, three main themes emerged, directly related to our research questions: (1) the relationship between emotional competence and EBN in different healthcare contexts, encompassing sub-themes of (i) impact on quality and safety of care, (ii) abilities in clinical reasoning and decision-making, (iii) influence on interprofessional collaboration in EBP implementation, and (iv) impact on professional performance in cultural and high-complexity contexts; (2) research methodologies used to study this relationship; and (3) main challenges and suggestions for integrating emotional competence and EBP in nursing practice. The findings related to the sub-themes of the first theme are presented in Table 2, Table 3, Table 4 and Table 5.

### 3.1. Impact of Emotional Competence on Quality and Safety of Care

Table 2 summarizes the findings related to the impact of emotional competence on the quality and safety of care. The studies included in this section highlight how emotional competence influences adherence to evidence-based protocols and improves patient outcomes, such as reducing infections and preventing falls.

**Table 2:** Impact of Emotional Competence on Quality and Safety of Care.

Author, Year (Citation)	Context	Main Results	Conclusion
Adams & Iseler, 2014 [34]	Hospital	Significant correlation between emotional competence and quality and safety of care indicators (prevention of Clostridium difficile and Staphylococcus aureus infections, reduction of falls with injuries, pressure ulcer assessments in intensive care)	Data Increasing emotional competence in the nursing team can improve the quality of care, suggesting that emotional competence facilitates adherence to evidence-based protocols and guidelines
Fujino et al., 2014 [35]	Hospital	Nurses with high emotional competence demonstrate greater involvement in professional development activities and exhibit better nursing performance, facilitating fast and effective reasoning	Emotional competence emerges as a critical facilitator of clinical performance, allowing nurses to establish interpersonal connections and make effective decisions in varying contexts

### 3.2. Emotional Competence Abilities in Clinical Reasoning and Decision-Making

Table 3 presents the findings related to the role of emotional competence in clinical reasoning and decision-making. The studies in this section demonstrate how emotional competence enhances nurses’ ability to manage emotions, communicate effectively, and make patient-centered decisions, even in ethically challenging situations.

**Table 3:** Emotional Competence Abilities in Clinical Reasoning and Decision-Making.

Author, Year (Citation)	Context	Main Results	Conclusion
Bahmanpour et al., 2018 [36]	Hospital	Emotional competence is an essential component of critical thinking in nursing, influencing structured clinical reasoning and holistic and evidence-based decision-making	Emotional competence contributes to emotion management, effective communication, and stress management, enabling agile and patient-centered responses
Hutchinson et al., 2017 [37]	Regional health service	Emotional competence manifests in clinical reasoning and decision-making through emotional self-awareness, emotion management, empathy, and social skills, influencing how nurses assess situations and interact with patients and colleagues	Emotional competence plays a crucial role in clinical reasoning and decision-making and should be integrated into training and professional development
McLemore et al., 2015 [40]	Care units for women needing abortions	Decision-making in abortion-related care involves a complex process of integrating moral, ethical, scientific, legal, social, political, and experiential information, where EI assists in managing emotions in ethically challenging contexts	Emotional competence facilitates evidence-based practice, functioning as a pillar of emotional and empathetic stability, allowing nurses to face highly emotionally charged situations
Zaki et al., 2018 [39]	Hospitalar	An emotional intelligence training program resulted in a significant improvement in the level of emotional competence and decision-making style among head nurses	Emotional competence positively influences decision-making styles in head nurses, reinforcing the importance of interventions for its development
Btoush et al., 2024 [38]	Hospital intensive care units	Positive correlation between emotional competence, self-efficacy, and clinical decisions; gender differences influence levels of self-efficacy and emotional competence	Intensive care nurses with higher emotional competence are more confident in their abilities (self-efficacy) and, consequently, make better clinical decisions, demonstrating the importance of investing in the development of these competencies

### 3.3. Emotional Competence and Interprofessional Collaboration in EBN Implementation

Table 4 explores the role of emotional competence in fostering interprofessional collaboration during the implementation of evidence-based nursing (EBN). The studies in this section highlight how emotional competence enhances communication, teamwork, and adaptability, facilitating the integration of EBP into routine care.

**Table 4:** Emotional Competence and Interprofessional Collaboration in EBN Implementation.

Author, Year (Citation)	Context	Main Results	Conclusion
Clarke et al., 2023 [41]	Hospital and community	Interprofessional collaboration is facilitated by emotional competence, through effective communication, understanding different perspectives, building interpersonal relationships, leadership, and mediation, being essential for EBP implementation	Emotional competence promotes a collaborative environment and facilitates EBP implementation, highlighting the need to create work environments that promote interprofessional collaboration
Hov et al., 2009 [42]	Nursing homes (urban and rural)	Emotional competence facilitates the adaptation of EBP to individual user needs and the specific context, allowing consideration of relevant emotional, cultural, and social aspects, empowering nurses to deal with the emotional challenges of their work	Emotional intelligence, through competencies such as empathy, emotional management, and social skills, is essential for the effective implementation of EBP in complex and emotionally demanding contexts, such as end-of-life care for the elderly

### 3.4. Emotional Competence and Professional Performance in Cultural and High-Complexity Contexts

Table 5 examines the impact of emotional competence on professional performance in culturally diverse and high-complexity healthcare contexts. The studies in this section demonstrate how emotional competence supports cultural adaptation, effective communication, and rapid decision-making, particularly in technologically advanced environments like telemedicine.

**Table 5:** Emotional Competence and Professional Performance in Cultural and High-Complexity Contexts.

Author, Year (Citation)	Context	Main Results	Conclusion
Al-Hamdan et al., 2016 [43]	Hospital	Positive and significant correlation between emotional competence and professional performance, influenced by cultural factors that shape the expression and management of emotions in the work environment	In the analyzed hospital context, emotional competence plays an important role in professional performance and, consequently, in EBP implementation. The ability to recognize, understand, and manage emotions, both one’s own and those of others, facilitates effective communication, interprofessional collaboration, informed clinical decision-making, and adaptation to changes. Emotional competence allows nurses to deal with the complexities of the hospital environment and consider cultural nuances in the application of EBP, resulting in better care for users
Williams et al., 2019 [44]	Hospital (intensive care units using telemedicine)	Emotional competence is essential for professional performance and for preventing failure to rescue in tele-ICU contexts. It facilitates effective communication through digital means, rapid decision-making under pressure, adaptation to new technologies and processes, and the management of cultural complexities and technology-mediated communication	While telemedicine offers significant benefits, it introduces new complexities that require a high level of emotional competence from nurses to ensure its effective implementation and the improvement of user care. Nurses’ emotional competence plays a fundamental role in maximizing the benefits of this technology, allowing for effective communication, rapid decision-making, and adaptation to new ways of working, even in cultural and high-complexity contexts

## 4. Discussion

This scoping review explored the relationship between nurses’ emotional competence and evidence-based nursing, addressing the following research questions: “What is the relationship between nurses’ emotional competence and evidence-based practice in different healthcare contexts?”, “Which research methodologies are most frequently used to study the relationship between emotional competence and evidence-based practice among nurses?”, and “What are the main challenges identified in the implementation of evidence-based practices that may be related to nurses’ emotional competence?”.

The 11 included studies, employing a mix of quantitative (e.g., cross-sectional, correlational) and qualitative (e.g., phenomenological, thematic analysis) methodologies, confirm the observation of Bulmer Smith et al. [45] regarding the scarcity of direct evidence linking nurses’ emotional competence with the adoption of EBP. However, as these authors suggest, understanding how emotions influence the use and application of knowledge is essential to comprehend the difficulties nurses face in integrating evidence-based practice into their daily routines.

The synthesis of findings revealed three main themes, directly related to our research questions: (1) the relationship between emotional competence and EBN in different healthcare contexts, encompassing sub-themes such as its impact on the quality and safety of care, clinical reasoning and decision-making, interprofessional collaboration, and professional performance in cultural and high-complexity settings; (2) the research methodologies used to study this relationship; and (3) the main challenges and suggestions for integrating emotional competence and EBP in nursing practice. These findings are relevant to nurses, nurse educators, healthcare organizations, and policymakers, as they highlight the crucial role of emotional competence in EBN.

### 4.1. Relationship Between Emotional Competence and EBN in Different Healthcare Contexts

The analyzed studies suggest that emotional competence plays an important role in clinical reasoning and the quality and safety of care provided. It appears to facilitate EBN by promoting clinical decision-making, empathetic communication, and adherence to best practices across various healthcare settings, ultimately improving patient outcomes.

Impact of Emotional Competence on Quality and Safety of Care: Adams and Iseler [34] found a significant correlation between nurses’ emotional competence and quality of care (QOC) indicators. Specifically, higher emotional competence was associated with better infection prevention, such as reducing cases of Clostridium difficile and Staphylococcus aureus, as well as improved patient safety outcomes, including fewer falls with injury. This correlation suggests that emotional competence facilitates adherence to evidence-based protocols and guidelines. It also supports the hypothesis that high emotional competence promotes better management of complex and stressful situations, fostering safer and more rigorous behaviors that contribute to error prevention. Fujino et al. [35] also linked high emotional competence to nurses’ overall performance, arguing that it promotes engagement in professional development and enhances clinical performance by enabling nurses to establish interpersonal connections and make quick, effective decisions in diverse contexts.

Role of Emotional Intelligence in Clinical Reasoning and Decision-Making: Bahmanpour et al. [36] revealed that emotional competence is an essential component of critical thinking in nursing, influencing clinical reasoning and holistic, evidence-based decision-making. Emotional competence supports emotion management, effective communication, and stress management skills, enabling agile and patient-centered responses. This aligns with evidence that clinical decision-making is more effective when both emotional context and evidence-based guidelines are considered, as demonstrated by Hutchinson et al. [37].

McLemore et al. [40] concluded that emotional intelligence (EI) is fundamental for nurses managing their own emotions and those of others in ethically challenging contexts, such as abortion-related care. The ability to regulate emotions and maintain empathy helps professionals make thoughtful and impartial decisions, essential for ethical and patient-centered care. This study exemplifies how EI can facilitate evidence-based practice by providing emotional stability in high-stress situations, where personal biases and social pressures may influence decision-making.

These findings reinforce that integrating EI into clinical reasoning models can enrich EBN, enabling more compassionate and ethically grounded decisions while improving professional and patient engagement. This balance allows nurses to act quickly in high-pressure contexts while maintaining a scientifically grounded and emotionally appropriate approach, as evidenced by Zaki et al. [39] and Btoush et al. [38], who associated high emotional competence with improved decision-making skills and clinical effectiveness.

Emotional competence and interprofessional collaboration in EBP implementation: Clarke et al. [41] documented that nurses with high emotional competence exhibit better collaboration and adaptability, facilitating EBN implementation, which often requires teamwork. These results suggest that emotional competence not only enhances individual performance but also fosters a collaborative environment, optimizing communication and receptiveness to practice changes. Hov et al. [42] corroborate these findings, stating that emotional competence promotes self-awareness and reflection, enabling nurses to identify areas for improvement and align their practice with the best evidence. Thus, individuals with high emotional competence tend to be more adaptable to new situations, a crucial characteristic for EBN implementation, which requires constant updating and adaptation to new evidence.

Emotional Competence in Cultural and High-Complexity Contexts: The impact of emotional competence on nurses’ professional performance must be considered within cultural contexts, particularly in countries like Jordan, where hierarchical structures and conservative norms shape workplace interactions.

Al-Hamdan et al. [43] demonstrated that high emotional competence facilitates cultural adaptation and the adoption of evidence-based practices. Using the Schwirian scale (1978), the study showed that nurses with higher emotional competence scores exhibited superior performance, suggesting that better emotional management can ease the adoption of evidence-based practices in culturally diverse settings.

Williams et al. [44] explored nursing practice in tele-ICU environments, where emotional competence facilitated effective digital communication, rapid decision-making under pressure, and adaptation to new technologies. The complexities of telemedicine require high emotional competence to ensure effective implementation and improved patient care. Nurses utilized EI competencies, such as empathetic communication and situational awareness, to perform proactive clinical interventions. Technological tools, such as audiovisual communications and decision support software, facilitated the integration of emotional and technical competencies, essential for EBN. This study reinforces that EI promotes technological integration and enhances collaboration, supporting EBN in high-complexity contexts.

### 4.2. Research Methodologies Used to Study the Relationship Between Emotional Competence and EBN

The studies included in this review employed diverse methodological approaches, reflecting the complexity of the relationship between emotional competence and EBN. While the results section detailed these methodologies, it is worth noting that the combination of quantitative and qualitative methods allowed for a comprehensive understanding of the phenomenon, reconciling measurable data with nurses’ subjective experiences.

Quantitative approaches: Quantitative studies with cross-sectional and correlational designs predominated, seeking to establish statistical associations between emotional competence and variables related to EBN, such as quality of care, professional performance, and clinical decision-making. For example, Adams and Iseler [34] used a multivariate linear regression analysis to examine the relationship between emotional competence and quality of care indicators, while Al-Hamdan et al. [43] applied Pearson’s correlation coefficient and multiple regression to investigate the relationship between emotional competence and work performance. Fujino et al. [35] also adopted a cross-sectional correlational approach to analyze the characteristics of nursing performance in nurses with high emotional competence and the influence of years of experience. Some studies, such as that of Zaki et al. [39], adopted a quasi-experimental approach to assess the impact of emotional competence development programs on decision-making styles. Btoush et al. [38] conducted a cross-sectional, descriptive, and correlational study that evaluated the relationship between emotional intelligence, self-efficacy, and clinical decision-making in intensive care nurses. To analyze the relationships between these variables, they used the Partial Least Squares (PLS) approach, a robust statistical technique suitable for complex models with multiple variables. The use of standardized instruments, such as the Mayer-Salovey-Caruso Emotional Intelligence Test (MSCEIT) [34], the Genos EI Assessment [43], the Wong Law Emotional Intelligence Scale (WLEIS) [38], and the EQS [35], was common among the quantitative studies, allowing for the comparison and generalization of results.

Qualitative approaches: The qualitative studies adopted diverse approaches, including interpretive phenomenology [41], hermeneutic phenomenology [42], thematic analysis [37], and content analysis [36]. McLemore et al. [40] used a qualitative descriptive approach, complemented by thematic analysis, to describe nurses’ decision-making in abortion-related care, using semi-structured interviews and the Atlas.ti software (version not specified; ATLAS.ti Scientific Software Development GmbH; https://atlasti.com; accessed on 15 January 2025) to assist in data analysis. Williams et al. [44] conducted an interpretive qualitative study that explored nursing interventions in tele-ICU to prevent rescue failures, using telephone, face-to-face, and videoconference interviews, with thematic analysis assisted by NVivo software (version not specified; NVivo software; QSR International, Melbourne, Australia; https://lumivero.com/; accessed on 15 January 2025). Hutchinson et al. [37] specifically explored how emotional intelligence abilities are used in clinical reasoning and decision-making, using individual semi-structured interviews conducted after the completion of an emotional intelligence training program and individual coaching sessions. The data analysis, performed by two independent researchers, used inductive thematic analysis, focusing on the identification of implicit and explicit ideas in the data, with the GENOS EI model (Genos International, https://www.genosinternational.com/emotional-intelligence/ accessed on 12 January 2025) providing a framework for the initial coding and thematic development. These studies focused on understanding nurses’ experiences and perspectives on the influence of emotional competence on their clinical practice and EBN implementation. Semi-structured interviews were the predominant data collection method, allowing for an in-depth exploration of the experiences and meanings attributed by the participants. For example, Clarke et al. [41] explored the experience of advanced nurses and obstetricians in interprofessional collaboration during EBN implementation, while Hov et al. [42] sought to understand the meaning of being a nurse in a nursing home for end-of-life patients.

The combination of quantitative and qualitative approaches, although not present in all individual studies, allowed for data triangulation and a more holistic understanding of the phenomenon. While the quantitative studies provided evidence on the associations between variables, the qualitative studies deepened the understanding of the mechanisms and processes underlying these relationships.

### 4.3. Main Challenges and Suggestions for the Integration of Emotional Competence and EBN in Practice

The third research question sought to identify the main challenges in EBP implementation related to nurses’ emotional competence. The analysis revealed several obstacles, as well as suggestions to overcome them.

Gaps in Emotional Intelligence Training: A key barrier identified is the insufficient focus on emotional intelligence training in nurses’ initial education [36]. This deficiency can compromise the development of essential competencies, such as self-awareness, emotional management, empathy, and social skills, which are fundamental for EBN application. The studies suggest the need for training programs that integrate emotional competencies with technical and scientific knowledge, preparing professionals for the complexities of clinical practice. Clarke et al. [41], Bahmanpour et al. [36], and Fujino et al. [35] support this perspective, emphasizing that an integrated training approach can optimize clinical performance and well-being, contributing to a more balanced and sustainable practice.

Organizational and Cultural Challenges: EBN implementation also faces organizational and cultural challenges. Resistance to changes in routines and care practices is common, particularly in hierarchical or culturally conservative contexts [41,43]. Emotional competence emerges as a facilitating factor for adaptation, promoting receptiveness to innovation and the implementation of new evidence. Work environments that foster interprofessional collaboration, providing adequate resources, time, and training, are essential to overcoming these barriers.

Cultural factors also play a significant role in the relationship between emotional competence and EBN. Al-Hamdan et al. [43] highlight the influence of cultural context on how emotions are expressed and managed in the workplace. Culturally sensitive training approaches that promote self-awareness and emotional management in line with local norms can be more effective in fostering emotional competence and EBN implementation.

Adaptation to New Technologies and Care Contexts: Adapting to new technologies and work processes, as observed by Williams et al. [44] in telemedicine contexts, represents an additional challenge. Emotional competence facilitates the integration of these new tools and processes, enabling nurses to manage resistance to change and effectively utilize available technologies. Specific training on virtual communication and stress management in remote support situations is crucial in this context.

Consideration of Individual Patient Needs: Hov et al. [42] emphasize the need to adapt EBN to individual patient needs and specific contexts, considering emotional, cultural, and social aspects. Emotional intelligence training is fundamental to improving adaptability and addressing these aspects in EBN implementation.

Intervention Suggestions: Based on the identified challenges, the studies propose several interventions, including the development of continuous training programs that integrate emotional intelligence competencies, the creation of collaborative work environments, and the implementation of culturally sensitive training approaches. Emotional intelligence training emerges as a cross-cutting element in these suggestions, reinforcing its importance for effective EBN implementation.

This scoping review has limitations. Our search, restricted to articles in English, Portuguese, French, and Spanish, may have excluded relevant studies in other languages. Although multiple databases were used, some relevant studies may not have been indexed. Study selection, performed by two independent reviewers, may contain some interpretation bias. Data extraction focused on mapping the literature rather than evaluating methodological quality, which limits the strength of conclusions about the relationship between emotional competence and EBN. However, we used the Joanna Briggs Institute (JBI) Critical Appraisal Checklist to assess the methodological quality of the included studies, and the findings from this assessment informed the discussion of the results, highlighting potential limitations in the evidence.

In addition to these limitations, it is important to acknowledge the methodological constraints of the included studies. Many relied on cross-sectional designs, which limit the ability to establish causal relationships between emotional competence and EBN outcomes. Furthermore, the heterogeneity in measurement instruments for emotional competence and EBN outcomes makes it difficult to compare findings across studies. These limitations underscore the need for future research to prioritize longitudinal designs and controlled intervention studies, which would provide stronger evidence for the causal impact of emotional competence on EBN implementation. Specific areas for further investigation include the role of emotional competence in diverse cultural contexts, the long-term impact of EI training programs on clinical outcomes, and the integration of emotional competence into nursing education curricula. Mixed-methods approaches, combining quantitative and qualitative data, would also offer a more comprehensive understanding of how emotional competence influences EBN in practice. Finally, while thematic synthesis is appropriate, it may lead to different interpretations of the data depending on the method used.

Based on the findings of this review, we propose the following practical recommendations to enhance the integration of emotional competence in nursing education and practice:

- Integrate Emotional Competence into Nursing Curricula: Nursing education programs should integrate emotional intelligence training into their curricula, focusing on self-awareness, emotion management, empathy, and social skills. This training should align with EBP principles to prepare nurses for the complexities of clinical decision-making.
- Develop Continuous Professional Development Programs: Healthcare organizations should offer ongoing training programs that combine emotional competence development with EBP skills. These programs should address cultural sensitivity, adaptability to new technologies, and stress management in high-pressure environments.
- Promote Interprofessional Collaboration: Work environments should foster interprofessional collaboration by providing resources, time, and training that support emotional competence and EBN implementation. This includes creating spaces for reflective practice and team-based learning.
- Implement Culturally Sensitive Approaches: Training programs should consider cultural factors that influence emotional expression and management, ensuring that emotional competence development is relevant to diverse healthcare contexts.

These recommendations aim to bridge the gap between emotional competence and EBN, ultimately improving patient outcomes and enhancing the well-being of nursing professionals.

## 5. Conclusions

This scoping review highlights the importance of integrating emotional competence into evidence-based nursing to enhance the quality, safety, and humanization of care. The findings suggest that nurses with higher emotional competence are better equipped to manage emotions, communicate effectively, and make patient-centered decisions aligned with EBN guidelines. Emotional competence facilitates the implementation of evidence-based practices in areas such as infection prevention, fall management, and adherence to protocols, even in emotionally demanding contexts. It also promotes structured clinical reasoning, interprofessional collaboration, and adaptability to new technologies, supporting EBP integration in diverse healthcare settings. By balancing technical and emotional competencies, emotional competence enables ethical and empathetic decision-making, particularly in complex situations.

However, the studies identified significant gaps in nurses’ training in emotional competencies, particularly during initial education. The integration of empathy, self-awareness, and emotional management development in initial and continuing education is essential to optimize EBN application, especially in challenging contexts. Interdisciplinary educational programs that address these dimensions can reduce barriers to implementing evidence-based practice, improve clinical performance and patient safety, and enhance professionals’ well-being. Such initiatives promote sustainable, patient-centered care and strengthen nurses’ resilience in the face of increasing care demands.

In summary, this review underscores the importance of integrating emotional competence into EBN to continuously improve nursing practice and promote ethical, safe, and patient-centered care. Investing in robust research and training interventions to develop emotional competence in nurses is essential to optimize EBN implementation and, consequently, enhance the quality of care. Future studies should explore this relationship in greater depth, to develop practice models that reconcile emotional competencies and evidence-based practices.

## Data Availability

Not applicable.

## Public Involvement Statement

No public involvement in any aspect of this research.

## Guidelines and Standards Statement

This manuscript was drafted against the PRISMA-ScR (Preferred Reporting Items for Systematic reviews and Meta-Analyses extension for Scoping Reviews. A complete list of reporting guidelines can be accessed via the EQUATOR network: https://www.equator-network.org/ (accessed on 24 January 2025).

## Abbreviations

The following abbreviations are used in this manuscript:

EBN	Evidence-based nursing
PRISMA	Preferred Reporting Items for Systematic Reviews and Meta-Analyses
EBP	Evidence-based practice
EI	Emotional intelligence
MSCEIT	Mayer–Salovey–Caruso Emotional Intelligence Test
TEIQue	Trait Emotional Intelligence Questionnaire
EQ-i	Bar-On Emotional Quotient Inventory
ESCI	Emotional and Social Competence Inventory
JBI	Joanna Briggs Institute
PRISMA-ScR	Preferred Reporting Items for Systematic Reviews and Meta-Analyses
	extension for Scoping Reviews
PCC	Population, Concept, and Context
QOC	Quality of care
MDPI	Multidisciplinary Digital Publishing Institute

## Appendix A

### Search Strategy

1. **CINAHL Complete & MEDLINE Complete (via EBSCO)**
  TI (nurs* OR “nurse practitioner*” OR “licensed practical nurse*”) AND (“emotional intelligence” OR “social intelligence” OR “emotional competence skills” OR “perceived emotional intelligence” OR “emotional competence”) AND (“evidence-based nursing” OR “evidence-based practice” OR “supported decision making” OR empowerment OR “decision making” OR “clinical competence” OR “clinical decision making” OR “advanced practice nurses” OR “Research-Based”) OR AB TI (nurs* OR “nurse practitioner*” OR “licensed practical nurse*”) AND (“emotional intelligence” OR “social intelligence” OR “emotional competence skills” OR “perceived emotional intelligence” OR “emotional competence”) AND (“evidence-based nursing” OR “evidence-based practice” OR “supported decision making” OR empowerment OR “decision making” OR “clinical competence” OR “clinical decision making” OR “advanced practice nurses” OR “Research-Based”) OR SU TI (nurs* OR “nurse practitioner*” OR “licensed practical nurse*”) AND (“emotional intelligence” OR “social intelligence” OR “emotional competence skills” OR “perceived emotional intelligence” OR “emotional competence”) AND (“evidence-based nursing” OR “evidence-based practice” OR “supported decision making” OR empowerment OR “decision making” OR “clinical competence” OR “clinical decision making” OR “advanced practice nurses” OR “Research-Based”)
2. **Nursing & Allied Health Collection: Comprehensive (via EBSCO)**
  (KW nurses OR AB nurs* OR AB “nurse practitioner*” OR AB “licensed practical nurse*”) AND (KW “Emotional Intelligence” OR AB “emotional intelligence*” OR AB “social intelligence*” OR AB “Emotional competence skills” OR AB “Perceived Emotional Intelligence” OR AB “Emotional competence”) AND (KW “Evidence-Based Nursing” OR KW “Evidence-Based Practice” OR AB “Supported decision making” OR AB “Empowerment” OR AB “Evidence-Based Practice” OR AB “evidence based nursing*” OR AB “Decision making” OR AB “Clinical competence” OR AB “Clinical decision making” OR AB “Advanced practice nurses” OR AB “evidence based*” OR AB “Research-Based”)
3. **Cochrane Central Register of Controlled Trials (via EBSCO)**
  (MH nurses OR AB nurs* OR AB “nurse practitioner*” OR AB “licensed practical nurse*”)) AND ((MH “Emotional Intelligence” OR AB “emotional intelligence*” OR AB “social intelligence*” OR AB “Emotional competence skills” OR AB “Perceived Emotional Intelligence” OR AB “Emotional competence”)) AND ((MH “Evidence-Based Nursing” OR MH “Evidence-Based Practice” OR AB “Supported decision making” OR AB “Empowerment” OR AB “Evidence-Based Practice” OR AB “evidence based nursing*” OR AB “Decision making” OR AB “Clinical competence” OR AB “Clinical decision making” OR AB “Advanced practice nurses” OR AB “evidence based*” OR AB “Research-Based”)
4. **MedicLatina**
  (KW nurses OR AB nurs* OR AB “nurse practitioner*” OR AB “licensed practical nurse*”) AND (KW “Emotional Intelligence” OR AB “emotional intelligence*” OR AB “social intelligence*” OR AB “Emotional competence skills” OR AB “Perceived Emotional Intelligence” OR AB “Emotional competence”) AND (KW “Evidence-Based Nursing” OR KW “Evidence-Based Practice” OR AB “Supported decision making” OR AB “Empowerment” OR AB “Evidence-Based Practice” OR AB “evidence based nursing*” OR AB “Decision making” OR AB “Clinical competence” OR AB “Clinical decision making” OR AB “Advanced practice nurses” OR AB “evidence based*” OR AB “Research-Based”)
5. **Psychology and Behavioral Sciences Collection**
  (SU “nurses” OR AB “nurse practitioner*” OR AB “licensed practical nurse*” OR AB “nurse*” OR AB “Nursing”) AND (SU “Emotional Intelligence” OR AB “Perceived Emotional Intelligence” OR AB “Emotional competence” OR AB “emotional intelligence*” OR AB “social intelligence*” OR AB “Emotional competence skills”) AND (SU “Evidence-Based Nursing” OR SU “Evidence-Based Practice” OR AB “Supported decision making” OR AB “Empowerment” OR AB “Evidence-Based Practice” OR AB “evidence based nursing*” OR AB “Decision making” OR AB “Clinical competence” OR AB “Clinical decision making” OR AB “Clinical nursing” OR AB “evidence based*” OR AB “Advanced practice nurses”)
6. **SCOPUS**
  (KEY (nurses) OR TITLE-ABS-KEY (“nurse practitioner*”) OR TITLE-ABS-KEY (“licensed practical nurse*”) OR TITLE-ABS-KEY (nurs*)) AND (KEY (“Emotional Intelligence”) OR TITLE-ABS-KEY (“Perceived Emotional Intelligence”) OR TITLE-ABS-KEY (“Emotional competence”) OR TITLE-ABS-KEY (“emotional intelligence*”) OR TITLE-ABS-KEY (“social intelligence*”) OR TITLE-ABS-KEY (“Emotional competence skills”)) AND (KEY (“Evidence-Based Nursing”) OR KEY (“Evidence-Based Practice”) OR TITLE-ABS-KEY (“Supported decision making”) OR TITLE-ABS-KEY (“Empowerment”) OR TITLE-ABS-KEY (“Evidence-Based Practice”) OR TITLE-ABS-KEY (“evidence based nursing*”) OR TITLE-ABS-KEY (“Decision making”) OR TITLE-ABS-KEY (“Clinical competence”) OR TITLE-ABS-KEY (“Clinical decision making”) OR TITLE-ABS-KEY (“Clinical nursing”) OR TITLE-ABS-KEY (“evidence based*”) OR TITLE-ABS-KEY ( “Advanced practice nurses”)) AND PUBYEAR > 1997 AND PUBYEAR < 2025
7. **Web of Science**
  1: (((AK = (nurs*)) OR AB=(nurs*)) OR AB = (nurse practi*)) OR AB = (licensed practical nurs*) 2: ((((((AK = (emotional intelligence)) OR AB = Emotional intelligence)) OR AB = (Perceived Emotional Intelligence)) OR AB = (Emotional competence)) OR AB = (emotional intelligence)) OR AB = (social intelligence)) OR AB = (emotional competence skills) 3: ((((((((((((AK=(Evidence-Based Nursing)) OR AK = (Evidence-Based Practice)) OR AK = (Supported decision making)) OR AB = (Supported decision making)) OR AB = (Evidence-Based Nursing)) OR AB=(Evidence-Based Practice)) OR AB = (Empowerment)) OR AB = (Decision making)) OR AB = (Clinical competence)) OR AB = (Clinical decision making)) OR AB = (Clinical nursing)) OR AB = (evidence based*)) OR AB = (Advanced practice nurs*) 4: #1 AND #2 AND #3
